# The Protective Effect of the Total Flavonoids of *Abelmoschus esculentus* L. Flowers on Transient Cerebral Ischemia-Reperfusion Injury Is due to Activation of the Nrf2-ARE Pathway

**DOI:** 10.1155/2018/8987173

**Published:** 2018-08-05

**Authors:** Yang Luo, Hong-Xin Cui, An Jia, Shan-Shan Jia, Ke Yuan

**Affiliations:** ^1^Zhejiang Agriculture and Forestry University, Lin'an 311300, China; ^2^College of Pharmacy, Henan University of Chinese Medicine, Zhengzhou 450046, China; ^3^College of Medicine, Huanghe S & T University, Zhengzhou 450063, China; ^4^Jiyang College, Zhejiang Agriculture and Forestry University, Zhuji 311800, China

## Abstract

*Abelmoschus esculentus* L. has favorable nutritional/medicinal features. We found the content of total flavonoids in flower extract to be the highest (788.56 mg/g) of all the different parts of *A. esculentus*; according to high-performance liquid chromatography, the quercetin-3-O-[*β*-D-glu-(1 → 6)]-*β*-D-glucopyranoside content was 122.13 mg/g. Protective effects of an extract of the total flavonoids of *A. esculentus* flowers (AFF) on transient cerebral ischemia-reperfusion injury (TCI-RI) were investigated. Compared with the model group, mice treated with AFF (300 mg/kg) for 7 days showed significantly reduced neurologic deficits, infarct area, and histologic changes in brain tissue, accompanied by increased contents of superoxide dismutase, whereas contents of nitric oxide and malondialdehyde decreased. AFF upregulated the expression of Nrf2, HO-1, and NQO1. These data suggest that AFF protects against TCI-RI by scavenging free radicals and activating the Nrf2-ARE pathway.

## 1. Introduction

Ischemic cardiovascular disease (also known as “ischemic stroke”) is the third leading cause of death and disability worldwide [1]. The number of patients suffering from cerebral ischemic disease worldwide has increased by 2 million per year, and the morbidity associated with this disease can affect young people [2].

At present, several of the synthetic drugs used for the treatment of transient ischemic attack have side effects. “Natural” medicines have good curative effects and few side effects. In addition, cerebral ischemic disease is an emergency, difficult to predict, and its pathogenesis is complex [3]. During reperfusion after a transient ischemic attack, a combination of oxidative stress and release of excitatory neurotransmitters causes irreversible damage, inflammation, and even apoptosis of nerve cells [4, 5]. Therefore, searching for natural products for protection and treatment of transient cerebral ischemia-reperfusion injury (TCI-RI) and exploring their mechanism of action are a rational approach.

Nuclear factor-E2-related factor 2 (Nrf2) is a key regulator of defense against endogenous antioxidants. Most genes encoding antioxidant enzymes have antioxidant response element (ARE) sequences in their promoter regions. Studies have demonstrated that the activation of the Nrf2-ARE pathway contributes to neuroprotection following ischemic injury [6–8].

*Abelmoschus esculentus* L. commonly known as “lady's fingers,” “okra,” or “bhindi” is an important vegetable crop cultivated in many countries [9, 10]. The fruits are beneficial to the digestive and immune systems due to the high content of glycoproteins and microelements and are used as food additives because of their antigastric acid, antifatigue, antioxidation, and anti-inflammation properties [11]. The seeds of *A. esculentus* are a good source of many high-quality proteins and unsaturated fatty acids and have anticancer, antidiabetes mellitus, and antihyperlipidemia properties [12–14]. The flowering period of *A. esculentus* is long, and the yield is high, but *A. esculentus* flowers wither rapidly, so they tend not to be studied. *A. esculentus* flowers are good sources of flavonoids and polysaccharides and are involved in modulation of the immune system [15]. However, studies on the protective effects of an extract of the total flavonoids of *A. esculentus* flowers (AFF) on TCI-RI and its mechanism of action are lacking.

Therefore, we explore the protective effect of AFF on TCI-RI and its potential mechanism.

## 2. Material and Methods

### 2.1. Materials

The reference samples of quercetin-3-O-[*β*-D-glu-(1 → 6)]-*β*-D-glucopyranoside (AFG-1), quercetin-3-O-[*β*-D-xyl-(1 → 2)]-*β*-D-glucopyranoside (AFG-2), and quercetin-4^″^-O-methy-3-O-*β*-D-glucopyranoside (AFG-3) at a purity of >98% were separated by our research team [16]. Rutin reference sample was purchased from China Pharmaceutical and Biological Products Testing Institute. *A. esculentus* flower, fruit, and seed samples were picked up in vegetable test base of Zhejiang Agricultural and Forestry University in 2016. Nitric oxide (NO), malondialdehyde (MDA), superoxide dismutase (SOD), and Coomassie Brilliant Blue kit were purchased from Nanjing Jiancheng Biological Technology Co. Ltd. Antibodies against Nrf2, heme oxygenase-1 (HO-1), NAD(P)H:quinone oxidoreductase-1 (NQO1), and *β*-actin were purchased from Wanlei Biological Technology Co. Ltd. Chloral hydrate was purchased from Zhejiang Academy of Medical Sciences. All the reagents were of analytical or HPLC grade.

### 2.2. Extraction and Purification of Total Flavonoids from *A. esculentus*

Fresh samples of the flowers, fruits, and seeds of *A. esculentus* were weighed (10 kg), dried at 40°C, crushed, and passed through a 60-mesh sieve. These powders were reextracted by ultrasonication thrice with 70% ethanol : water at a 1 : 30 ratio (*w* : *v*) for 30 min each at room temperature. The extracts were combined and concentrated into a paste using a rotary evaporator. Then, the concentrated solution was extracted with ethyl acetate to remove fat-soluble components. The remaining extract was added to a column with resin (Diaion HP-20, Mitsubishi, Japan). The resin was washed with distilled water to remove proteins, polysaccharides, and other water-soluble impurities. Then, the eluate was collected with 50% methanol and dried by a rotary evaporator at 50°C. Powdered extracts of the total flavonoids of the flowers (258.8 g), fruits (186.3 g), and seeds (160.6 g) of *A. esculentus* were obtained and stored at 4°C.

### 2.3. Determination of the Composition of Total Flavonoids in Extracts

Each sample extract (10.0 mg), AFG-1 (31.6 mg), AFG-2 (3.25 mg), AFG-3 (5.08 mg), and rutin (10.0 mg) were dissolved in methanol and made up to 10 mL to provide samples and standard solutions. We measured the contents of total flavonoids using an AlCl_3_-colorimetric assay [17]. The absorbance was measured at 510 nm, and the content was expressed as milligram rutin equivalent per gram dry weight (mg RE/g DW). All samples were assayed thrice.

AFG-1, AFG-2, and AFG-3 contents were analyzed on a high-performance liquid chromatography (HPLC) system (2695; Waters, Milford, MA, USA) with a photodiode array detector (2996; Waters) under specific HPLC conditions: SunFire C18 column (4.6 mm × 250 mm, 5.4 *μ*m), column temperature = 28°C, flow rate = 0.8 mL/min, mobile phase = methanol (solvent A) : 0.1% phosphoric acid water (solvent B), and ratio of gradient elution, 47 : 53. These solutions were determined at an absorbance of 255.6 nm with sample feeding of 10 *μ*L. Identification of unknown peaks was based on comparison of the retention times with those of known standards.

### 2.4. Animal Experiments

All procedures were approved by the Committee on the Ethics of Animal Experiments at Zhejiang Agriculture and Forestry University (Zhejiang, China).

Male Kunming mice (18–22 g) were purchased from the Animal Experiment Center of Zhejiang Academy of Medical Sciences (Zhejiang, China; number SC 2008-3344). Before experimentation, all mice were maintained in a well-ventilated environment (23-24°C; humidity, 56–59%) with a 12 h light-dark cycle and had free access to food and water for 1 week.

**Figure 1:**
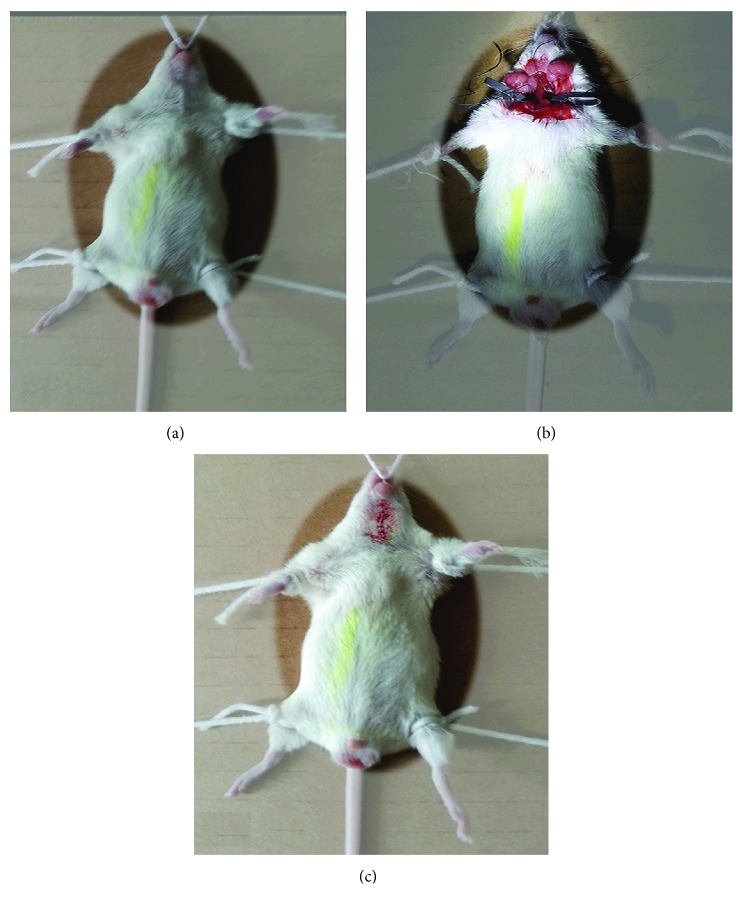
The process of TCI-RI operation. (a) The mouse was narcotized and fixed. (b) The middle neck of the mouse was cut, and the bilateral carotid artery was tied with thread and clamped with the arterial clip for 30 min. (c) After removal of the arterial clip and the line, the mouse wound was sutured and the mouse was reperfused for 24 h.

Mice (*n* = 75) were divided randomly into five groups, normal group (sham operation), model group, as well as high (300 mg/kg), medium (150 mg/kg), and low (75 mg/kg) AFF dose groups. 300 mg/kg has proven to be safe [18]. Mice in normal and model groups were given an equal volume of water, and in the other groups, the corresponding amounts of AFF were given once daily for 7 days. One hour after the final administration, mice in model and AFF groups were anesthetized (3.5% chloral hydrate, i.p.) and placed on a mouse fixator. Creation of the TCI-RI model is shown in Figure 1. The neck was disinfected with 75% alcohol. A midline incision was made, and skin was separated bluntly to allow exposure of bilateral common carotid arteries. Using an arterial clip, blood flow to bilateral common carotid arteries was blocked for 30 min. Subsequently, the arterial clip was loosened to recover this blood supply and the incision was sutured. In the normal group, bilateral common carotid arteries were not blocked and only suturing of the incision was done. After 24 h of reperfusion, the neurologic damage was evaluated. Then, 10 survived mice in each group were sacrificed, and their brain tissues were removed rapidly and stored at 20°C.

### 2.5. Survival and Neurologic Function Score

After TCI-RI for 24 h, the survival rate in each group was determined as the ratio of the number of survived mice to the total number of mice. An evaluator blinded to the treatment protocol undertook neurologic scoring as described by Longa et al. [19]. The scoring criteria are the following: 0 = no neurological deficits, 1 = failure to extend contralateral forepaw fully, 2 = circling to paretic side, 3 = falling to contralateral side, and 4 = did not walk spontaneously and has a depressed level of consciousness.

### 2.6. Evaluation of the Infarct Area

The brain tissues in each group (*n*_1_ = 3) were obtained in a random manner. Then, the cerebrums were cut into five coronal sections of thickness 2 mm. They were incubated immediately in 2% 2,3,5-triphenyltetrazolium chloride (TTC) solution at 37°C for 30 min in the dark and fixed in 10% formalin. After that, the stained slices were photographed using a camera (EOS30, Canon, Japan). The infarct area in each section was calculated using an image analyzer (Image-Pro Plus 6.0). The percentage of the infarct area was calculated using the following formula:

(1) $$\begin{array}{c} Infarct\;area\;\left(\%\right)=\frac{total\;infarct\;area}{total\;section\;area}\times 100. \end{array}$$

### 2.7. Histopathology

The whole brain tissues of each group (*n*_2_ = 3) after reperfusion for 24 h were fixed with 4% paraformaldehyde for 24 h, stained with hematoxylin and eosin (H&E), and then observed with an optical microscope (BX20, Olympus, Tokyo, Japan).

### 2.8. TUNEL Assay

The whole brain tissues of each group (*n*_2_ = 3) after reperfusion for 24 h were fixed with 4% paraformaldehyde for 24 h, regularly embedded in paraffin, sectioned at a thickness of 4 *μ*m, deparaffinized, stained with terminal deoxynucleotidyl transferase-mediated (dUTP) nick end labeling (TUNEL) reagents and DAPI solution, washed, and then photographed using a fluorescence microscope (Nikon Eclipse C1, Nikon, Japan). The positive cells (green spots) were identified and counted by an investigator blinded to the grouping. Three mice and 10 regions of the fluorescent images were used to obtain the apoptotic cell data.

### 2.9. Biochemical Analyses of Brain Tissue

The brain tissues in each group (n_3_ = 4) were homogenized with nine-fold physiological saline. The 10% homogenate was centrifuged at 3000 rpm for 10 min at room temperature. The 10% supernatants (100 *μ*L) were made into 2% concentration with cold physiological saline (400 *μ*L) to determine the protein content with Coomassie Brilliant Blue kit (standard solution: 0.563 g/L embryonic bovine serum BSA). The 10% supernatants were used to determine the contents of nitric oxide (NO), superoxide dismutase (SOD), and malondialdehyde (MDA) with the commercially available kits, and their results were expressed as equivalent per gram protein concentration.

### 2.10. Western Blotting

To analyze protein expression, brain tissue homogenates (*n*_3_ = 4) were centrifuged at 14,000 rpm for 30 min at 4°C to obtain total protein. Protein content was quantified using a bicinchoninic acid protein assay kit. Equal amounts of protein (50 *μ*g per lane) were resolved on 12% polyacrylamide gels, transferred to polyvinylidene fluoride (PVDF) membranes (Millipore, Marlborough, MA, USA), and probed with primary antibodies against Nrf2, HO-1, NQO1, and *β*-actin. PVDF membranes were washed thrice for 10 min each and incubated for 2 h at 4°C with horseradish peroxidase-conjugated secondary antibody (anti-rat). Proteins were visualized using an enhanced chemiluminescence detection system (Amersham Pharmacia, Piscataway, NJ, USA).

### 2.11. Statistical Analysis

Data were analyzed by one-way ANOVA with Duncan's test and intergroup comparison using SPSS statistical software (SPSS 19.0 Inc., Chicago, IL, USA) and expressed as mean ± standard deviation (SD). *P* values below 0.05 were considered statistically significant.

## 3. Results and Discussion

### 3.1. Contents of Total Flavonoids and Flavonoid Glycosides in Different Parts of *A. esculentus*

The fruits, seeds, roots, stems, leaves, and flowers of *A. esculentus* are used widely as traditional medicines in China because of their anticancer and anti-inflammatory effects [20]. It has been reported that the flowers, fruits, and seeds of *A. esculentus* are good sources of flavonoids and that the total content of flavonoids is different in different parts of *A. esculentus*.

**Figure 2:**
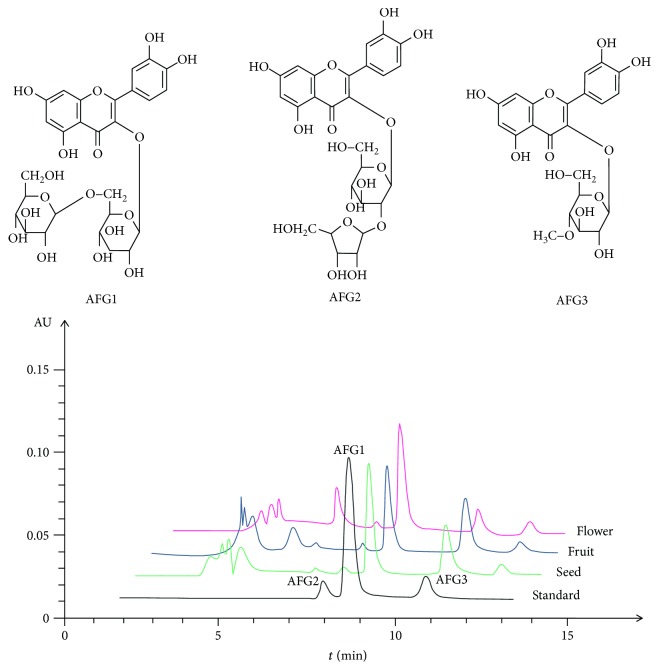
The structures and HPLC chromatogram of AFG-1, AFG-2, and AFG-3 in the flower, fruit, and seed of *A. esculentus*.

The total content of flavonoids in flower extract was highest in different parts of *A. esculentus*, and the highest amount was 788.56 mg/g (Table 1). The three flavonoid glycosides, AFG-1, AFG-2 and AFG-3, the structures of which are shown in Figure 2, were the main components in different parts of *A. esculentus* (Figure 2). In AFF, the AFG-1 content was 122.13 mg/g. Therefore, the potential protective effect of AFF upon TCI-RI was investigated.

### 3.2. Effect of AFF on the Survival Rate and Neurologic Damage

Transient cerebral ischemia is associated with high mortality and morbidity. Transient cerebral ischemia changes the cerebral ultrastructure and induces hypoxia and ischemia in the middle cerebral artery, which damages the nervous system [6, 21]. Only 52.6% of mice in the model group survived (Table 2). After AFF treatment, the survival rate was improved significantly depending on the AFF dose (*P* < 0.05). According to the observation of behavior and neurologic scores, mice in the normal group (which did not suffer damage to the nervous system) could move normally. The score of the model group was significantly different from that of the normal group (*P* < 0.01); the contralateral forepaw could not be extended forward or circled around, and athletic ability was weakened (neurologic score = 2.8 ± 0.79). The neurologic scores of the AFF (300 and 150 mg/kg) groups were significantly higher than those of the model group (*P* < 0.01), and mice did not fall to one side or suffer dyskinesia.

These results suggested that AFF could protect the nervous system from the effects of cerebral ischemic attack. Studies have shown that flavonoids have antioxidant functions [22]. Yuan et al. found that the total flavonoid extracts of flowers, fruits, leaves, and seeds all have free radical scavenging activity and antioxidant capacity and the free radicals scavenging capacity of AFF is relatively strong *in vitro* [23]. Neural cells in the brain have been more vulnerable to oxidative stress because they have high oxygen consumption and contain high levels of unsaturated fatty acids, essential prooxidants for lipid peroxidation, and low levels of antioxidant defense capacities [24]. Thereby, AFF scavenged free radicals to protect nerve cells from oxidative damage.

### 3.3. Effect of AFF on the Cerebral Infarction Area

Transient cerebral ischemia does not necessarily result in disability or death if timely return of oxygen and blood supply is initiated. Most cases of ischemic cardiovascular disease are caused by more severe reperfusion injury due to prolonged ischemia or hypoxia in the brain and heart [25, 26]. In patients with cerebral infarction, the infarct area of the middle cerebral arterial trunk is 82.12% [27]. Therefore, we used a method based on occlusion of the internal carotid artery to cause TCI-RI in mice, which results in symptoms similar to those of cerebral ischemia in humans.

**Figure 3:**
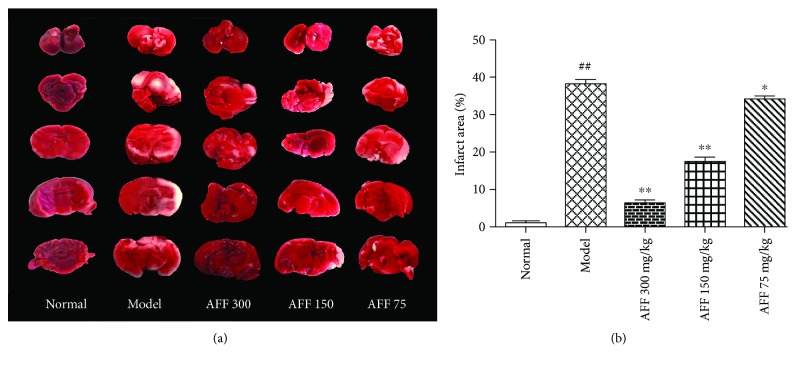
Effect of AFF on infarct areas in mice (*n*_1_ = 3) after TCI-RI. (a) Representative brain sections of TTC staining. The infarct areas were white. (b) Quantitative results of the infarction area. Values are mean ± SD. ^∗^ *P* < 0.05 and ^∗∗^ *P* < 0.01 versus the model group; ^##^ *P* < 0.01 versus the normal group.

Brain tissues were stained by TTC (Figures 3(a) and 3(b)), with red regions indicated normal tissue and white regions representing infarction. Compared with the normal group, mice in the model group had obvious cerebral infarction (39.13% ± 1.49). Pretreatment with AFF (300 and 150 mg/kg) reduced the cerebral infarct area markedly (*P* < 0.05). The results suggested a neuroprotective effect of AFF on TCI-RI mice. After subjection to cerebral ischemia and reperfusion, aerobic respiration gets compromised and the imbalance of Ca^2+^, Na^+^, and ADP ion homeostasis in the nerve cells stimulates the excessive production of mitochondrial oxygen radical [28]. Abnormal production of free radicals increases stress on cellular structures and damages intracellular macromolecules, such as lipids, proteins, and nucleic acids, leading to inactivation of enzymes, destruction of cell membranes, and dysfunction and eventually leading to death of brain tissue cells and excitotoxicity to aggravate the cerebral infarction area [29]. Elevated levels of lipid peroxidation and cytotoxins caused by oxidative stress after ischemia disrupt the tight junctions of endothelial cells, accompanying with increasing permeability of the blood-brain barrier (BBB) and worsening of the infarct severity [30]. Members of the flavonoid family have been reported to transverse BBB [31]. In AFF treatment groups, due to disruption of BBB after the TCI-RI event, AFF accumulated in TCI-RI regions improved neurologic function score and reduced the brain tissue infarct area through antioxidant activity, thereby promoting functional recovery of nerve cells. He et al. had demonstrated that Danhong injection promotes the recovery of neurological function with cerebral infarction by its antioxidant activities [32]. The previous study reported that flavonoids can also improve neurological function and protect brain tissue from cerebral ischemia-reperfusion injury by inhibiting oxidative stress to reduce the damage of the brain microvascular endothelial cell barrier and BBB function [6, 33].

### 3.4. Histopathology

**Figure 4:**
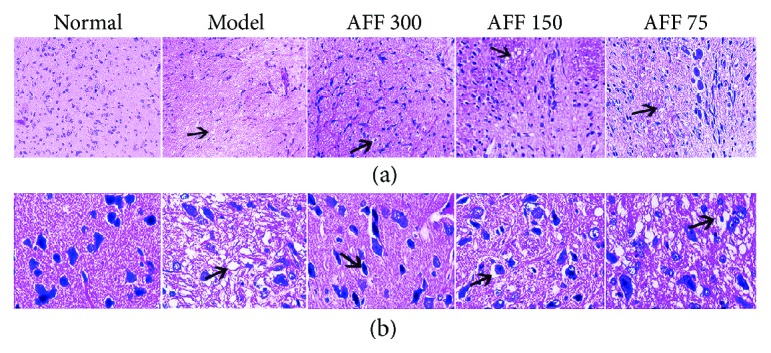
Effects of AFF on cortical area histopathologic changes in the brain of mice (*n*_2_ = 3) stained with H&E (×200; ×400). (a) HE-stained cerebral cortex of TCI-RI brain (×200). (b) HE-stained cerebral cortex of TCI-RI (×400). The arrows showed a gradual improvement on cellular edema and atrophic nucleolus.

Transient cerebral ischemia and hypoxia resulted in damage or necrosis of brain tissue accompanied by complex histopathologic changes [34]. Different lesions were observed according to the duration of ischemic insult. Upon ischemia for 30 min, the main damage was to neuronal cells [35]. After the whole brain tissue had been stained with H&E, histopathologic changes were clearly visible (Figures 4(a) and 4(b)). Compared with the normal group, cerebral cortical cells in the model group showed edematous cells, nucleolus atrophy, or cell loss, which were associated with histopathologic changes. Pretreatment with AFF for 7 days prevented neuronal cells from the damage induced by TCI-RI, and the high-dose group had the best effect.

### 3.5. Effect of the AFF on Cellular Apoptosis Detected by TUNEL Assay

**Figure 5:**
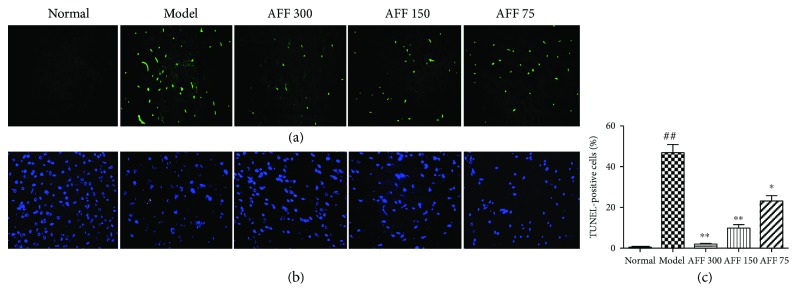
Effects of AFF on cortical area cellular apoptosis in the brain of mice (*n*_2_ = 3) with TUNEL assay (×400). (a) TUNEL-stained cerebral cortex of TCI-RI brain, the green fluorescence represented the apoptotic cells. (b) DAPI-stained cerebral cortex of TCI-RI. (c) Quantitative data on TUNEL-positive cells in cerebral cortex were obtained. Values are mean ± SD. ^##^ *P* < 0.01 versus the normal group; ^∗^ *P* < 0.05 and ^∗∗^ *P* < 0.01 versus the model group.

Abnormal formation of reactive oxygen species after reperfusion is one of the major factors inducing apoptosis in neuronal cells [36]. TUNEL staining was used to detect apoptotic cells on the basis of DNA fragmentation. The TUNEL-positive cells emitted green fluorescence after fluorescein labeling (Figure 5(a)). The normal group had no TUNEL-positive cells, and apoptotic cells in the model group increased significantly compared with the normal group. After AFF treatment (150 and 300 mg/kg BW), TUNEL-positive cells had significantly reduced when compared with the model group (Figure 5(c), *P* < 0.01). AFF had the potential action to protect the brain from TCI-RI damage by decreasing apoptotic cells. Zhang et al. found that pretreatment of flavonoid-rich extract from *Rosa laevigata* Michx fruit markedly inhibited neuron apoptosis by its antioxidant properties after TCI-RI [37].

### 3.6. Effect of AFF on the Contents of Protein, NO, SOD, and MDA in Brain Tissue

**Figure 6:**
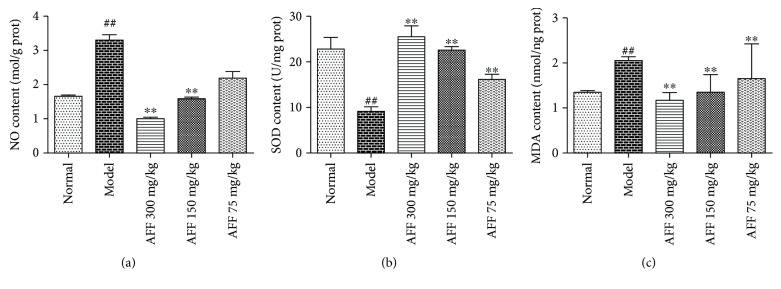
Effects of AFF on NO (a), SOD (b), and MDA (c) content in the brain tissue of mice (n_3_ = 4) after TCI-RI. Values are mean ± SD. ^##^ *P* < 0.01 versus the normal group; ^∗∗^ *P* < 0.01 versus the model group.

Several studies have shown that TCI-RI can cause severe reperfusion injury and produce a series of cascade reactions: energy depletion, oxidative stress with release of large amounts of free radicals, activation of apoptosis-related genes, calcium overload, release of excitatory neurotransmitters, and inflammation [38]. Oxidative stress is a major cause of secondary injury after TCI-RI. If brain tissue is subjected to ischemic stimulation, oxidative stress causes an imbalance of oxidants and antioxidants in tissue cells and produces excess reactive oxygen species (ROS) [39]. The latter damage the structure and function and inactivate enzymes within mitochondria, resulting in reduced production of adenosine monophosphate (ATP) within them [40]. Intracellular deficiency of ATP reduces the pH, inhibits the activity of Na^+^/Ca^2+^ exchange proteins, increases intracellular levels of Na^+^ and Ca^2+^, and reduces levels of K^+^. These ion disorders damage brain cell defense systems, resulting in the apoptosis or death of nerve cells [6, 28]. Ca^2+^ overload increases the expression of nitric oxide synthase (NOS) and Ca^2+^-dependent proteases. Then, NOS degrades glutamate to produce the nontraditional neurotransmitter NO. Subsequently, xanthine dehydrogenase is transformed to xanthine oxidase, which increases the levels of NO and oxygen free radicals [39, 41]. NO has complex roles in many diseases, including inhibition of mitochondrial function, and has toxic responses to induce cell death [42, 43]. The determination of the protein concentration in the tissue homogenate supernatant is not of a direct clinical value and used for the calculation of the relative content of other biochemical parameters [6, 37, 44]. The NO content increased markedly in mice of the model group compared with those in the normal group, and it was reduced significantly in AFF groups (300 and 150 mg/kg) compared with the model group; these differences were significant (Figure 6(a), *P* < 0.05).

The enzyme SOD scavenges superoxide anion radicals *in vivo* and can scavenge ROS, including superoxide anion radicals [45]. Decreased activity of SOD can cause massive accumulation of free radicals in brain tissue, which induces the lipid peroxidation of phospholipids and unsaturated fatty acids in cell membranes [6]. Then, levels of the final product of lipid peroxidation, MDA, increase accordingly, resulting in destruction of the structure and function of cell membranes and damage to neurons [46, 47]. Due to the scavenging of oxygen free radicals, SOD content is reduced and MDA content is increased after TCI-RI. Therefore, measuring the content of SOD and MDA can, indirectly, reflect the ability of the body to scavenge free radicals and the degree of damage caused by these chemicals [48]. Compared with the normal group, the SOD level in the brain tissue of mice in the model group was reduced significantly by 59.7% (*P* < 0.01) whereas the MDA content was increased by 51.9%. Pretreatment with AFF protected mice from TCI-RI, and levels of SOD and MDA were increased significantly and decreased, respectively (Figures 6(b) and 6(c), *P* < 0.01).

### 3.7. Effect of AFF on the Expression of Nrf2, NQO1, and HO-1

TCI-RI is a very complex pathologic process, so the mechanism is also complex. Several studies have shown that oxidative stress is one of the major causes of TCI-RI. Mechanisms of antioxidant stress include direct scavenging of free radicals as well as indirect antioxidant activity through modulation of the pathways involved in the expression of cytoprotective enzymes and molecules [7]. Chen et al. [49] reported that the extent of scavenging of the DPPH radical of flavonoids in *A. esculentus* flowers was greater than that of vitamin C at the same concentration. The inducible Nrf2-ARE pathway helps to regulate the expression of phase II-detoxifying and antioxidant enzymes. In a normal physiologic environment, Nrf2 is in the cytoplasm bound to its “natural restrainer,” Kelch-like ECH-associated protein 1 (Keap1), which induces the ubiquitination and constitutive degradation of Nrf2. If subjected to oxidative stress, Nrf2 dissociates from Keap1 and moves to the nucleus. Here, it binds to small musculoaponeurotic fibrosarcoma (Maf) protein to form a heterodimer and recognizes the appropriate ARE sequence to promote transcription of the antioxidant genes *SOD*, *HO-1*, and *NQO1* [8, 50–52].

**Figure 7:**
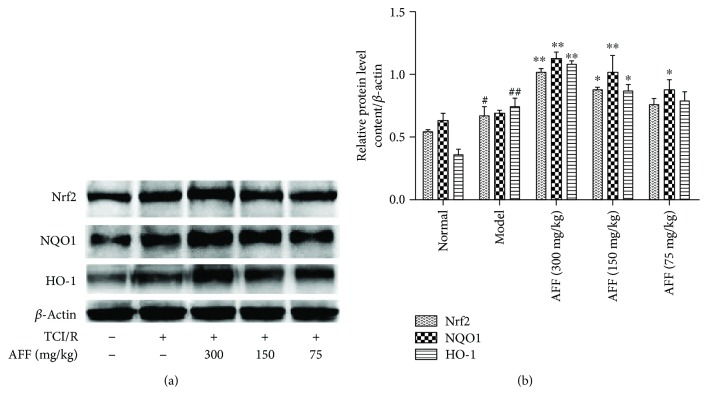
Effects of AFF on the expression level of Nrf2, NQO1, and HO-1 (*n*_3_ = 4). (a) The expression levels of Nrf2, NQO1, and HO-1 were analyzed by Western blotting. (b) Quantitative results of the expression levels. Values are mean ± SD. ^∗^ *P* < 0.05 and ^∗∗^ *P* < 0.01 versus the model group; ^#^ *P* < 0.05 and ^##^ *P* < 0.01 versus the normal group.

**Figure 8:**
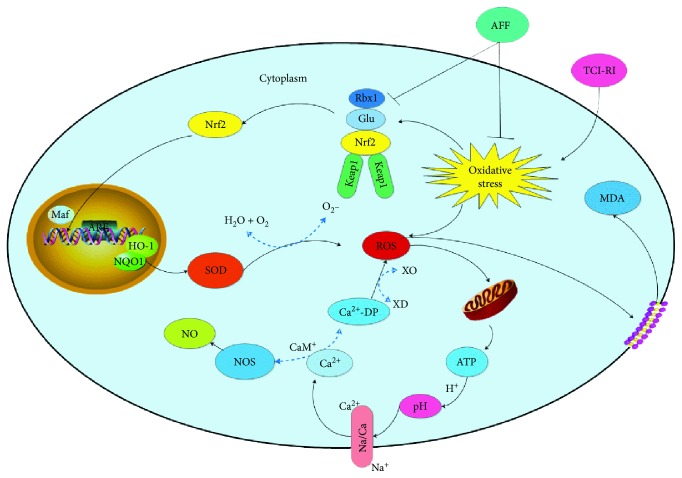
Mechanism of TCI-RI and protective effect of AFF on TCI-RI in nerve cells. AFF directly scavenged free radicals and indirectly activated the Nrf2-ARE pathway to enhance the expressions of antioxidase NQO1 and HO-1 and increase SOD content, against oxidative stress, which significantly reduced the content of NO, MDA, and protected TCI-RI.

Previous studies had demonstrated that total flavonoids have a protective effect on PC12 cells and through PC12 cells; it can be visually seen that Nrf2 is transferred from the cytoplasm to the nucleus when exposed to oxidative stress [53]. Huang et al. [54] found that the addition of HO-1 inhibitors (ZnPP) significantly reduces the protective effect on PC12 cells. Wu et al. [7] showed that mice lacking *Nrf2* are more susceptible to oxidative stress. Therefore, we investigated if AFF has a neuroprotective role by inducing the Nrf2-ARE pathway in TCI-RI *in vivo*. Compared with the model group, the expression of Nrf2, NQO1, and HO-1 in AFF-treated mice was upregulated significantly (Figures 7(a) and 7(b), *P* < 0.01). These results corresponded with the study [8]. Hence, the underlying molecular mechanism of the therapeutic effects of AFF on cerebral ischemic stroke was its antioxidant activity and modulation of the Nrf2-ARE pathway in response to oxidative stress (Figure 8). Nrf2 is a promising therapeutic target for defense against oxidative stress in stroke, and AFF will be an excellent medicine to protect against TCI-RI by activating the Nrf2-ARE pathway.

## 4. Conclusions

Our study demonstrated that AFF had protective effects against TCI-RI possibly by direct (scavenging free radicals) and indirect (activating the neuronal Nrf2-ARE pathway to modulate damage by oxidative stress) actions. This research provides a theoretical basis for the development of AFF as a functional food and its therapeutic effects on ischemic cardiovascular diseases.

## Figures and Tables

**Table 1:** Results for the determination of three kinds of flavonoid glycosides and total flavonoids in different parts extract of *A. esculentus*.

Part	AFG-1 (content/mg/g DW)	AFG-2 (content/mg/g DW)	AFG-3 (content/mg/g DW)	Total flavones (content/mg/g DW)	Powder yield
Flower	122.13	9.54	16.86	788.56	2.59%
Seed	53.16	3.01	28.06	627.04	1.86%
Fruit	35.96	2.66	25.59	520.83	1.61%

**Table 2:** Effect of AFF on the survival rate and neurologic score in mice subjected to TCI-RI.

Group	Dose (mg/kg/D)	*n*	*n* (survived mice)	Survival rate (%)	Neurologic score
Normal	0	10	10	100	0
Model	0	19	10	52.6	2.8 ± 0.79^##^
AFF (300 mg/kg)	300	12	10	83.3	1.2 ± 0.63^∗∗^
AFF (150 mg/kg)	150	16	10	62.5	1.5 ± 0.53^∗∗^
AFF (75 mg/kg)	75	18	10	55.5	1.9 ± 0.74^∗^

Values are mean ± SD. ^∗^ *P* < 0.05 and ^∗∗^ *P* < 0.01 versus the model group; ^##^ *P* < 0.01 versus the normal group.

## Data Availability

The data used to support the findings of this study are included within the article.

## Additional Points

*Chemical Compounds*. Rutin (PubChem CID: 5280805), 2,3,5-triphenyltetrazolium chloride (PubChem CID: 9283), and chloral hydrate (PubChem CID: 2707) are the chemical compounds used in the study.

## Supplementary Materials

Supplementary Materials The corresponding experimental data associated with this article can be seen from the supplementary materials' files.
